# Supplementary material for: Time-Driven Activity-Based Costing for Capturing the Complexity of Healthcare Processes: The Case of Deep Vein Thrombosis and Leg Ulcers
Source: Int J Environ Res Public Health. 2023 May 13;20(10):5817. doi: 10.3390/ijerph20105817 (PMC10218671; doi:10.3390/ijerph20105817)
Supplement: Supplementary file 1 [file ijerph-20-05817-s001.zip › Supplementary Table S1.pdf]

Supplementary Table S1. Study characteristics for standard of care.

Study	Study type	Country	Type of therapy	FUP (months)	N pts	N ulcers	% healed	Healing time (days)	N recurrences
Ivins 2020 [1]	Multi-center case series	Galles	3M Coban 2 Lite compression system	4	30	30	20.00%	112	-
Jull 2020 [2]	Pragmatic parallel group randomized controlled trial	New Zealand	Usual care	6	72	72	44.44%	159	4
Nunes 2019 [3]	Open, randomized, controlled clinical trial	Brazil	Unna boot	6	14	14	71.43%	112	-
			Cellulose membrane followed by Unna boot	6	14	14	64.29%	140	-
Tiwary 2020 [4]	Prospective study	India	Usual care (compression dressing)	12	60	64	93.75%	168	10

1. Ivins, N.; Jones, N. Two-Layer Reduced Compression System for Lower Limb Wounds: A Non-Comparative Evaluation. *Br. J. Community Nurs.* **2020**, *25*, S10–S16, doi:10.12968/bjcn.2020.25.Sup4.S10.
2. Jull, A.; Wadham, A.; Bullen, C.; Parag, V.; Weller, C.; Waters, J. Wool-Derived Keratin Dressings versus Usual Care Dressings for Treatment of Slow Healing Venous Leg Ulceration: A Randomised Controlled Trial (Keratin4VLU). *BMJ Open* **2020**, *10*, e036476, doi:10.1136/bmjopen-2019-036476.
3. Nunes, C.A. de B.; Melo, P.G.; Malaquias, S.G.; Amaral, K.V.Á.; Alves, G.R.; Meira, A.A.; Cardoso, A.L.; Pereira, L.V.; Bachion, M.M. Effectiveness of Two Bundles in Venous Leg Ulcer Healing: A Randomized Controlled Trial. *J. Vasc. Nurs. Off. Publ. Soc. Peripher. Vasc. Nurs.* **2019**, *37*, 232–245, doi:10.1016/j.jvn.2019.09.004.
4. Effect of Four-Layer Dressing on Venous Ulcer - Acta Phlebologica 2020 December;21(3):36-41 Available online: https://www.minervamedica.it/en/journals/acta-phlebologica/article.php?cod=R43Y2020N03A0036 (accessed on 25 July 2022).
