# Supplementary material for: Time-Driven Activity-Based Costing for Capturing the Complexity of Healthcare Processes: The Case of Deep Vein Thrombosis and Leg Ulcers
Source: Int J Environ Res Public Health. 2023 May 13;20(10):5817. doi: 10.3390/ijerph20105817 (PMC10218671; doi:10.3390/ijerph20105817)
Supplement: Supplementary file 1 [file ijerph-20-05817-s001.zip › Supplementary Table S2.pdf]

Supplementary Table S2. Study characteristics for endovascular treatment.

Study	Study type	Country	FUP (months)	N pts	N stents	Procedural success	N ulcers	% ulcers healed	mean healing time (months)	N recurrences
Lim 2020 [1]	Multicenter retrospective cohort study	Singapore	5.8	87	115	100%	43	95.3%	5.6	-
Zhou 2021 [2]	Retrospective study	China	28.8	127	127	100%	20	100%	-	-
Lugli-Longhi-Maleti	Real-world data	Italy	41	88	199	94.5%	88	81%	2	20

1. Lim, M.N.H.H.; Damodharan, K.; Chan, S.L.; Toh, M.R.; Yap, C.J.Q.; Chong, T.T.; Tang, T.Y. Endovascular Deep Vein Stenting of Symptomatic Post-Thrombotic and Non-Thrombotic Iliac Vein Stenotic Lesions: A Multicentre Cohort Experience from Singapore. *Ann. Acad. Med. Singapore* **2020**, *49*, 551–560.
2. Zhou, Y.; Guan, Y.; Xue, M.; Zheng, X.; Chen, X. Clinical Outcomes of Stenting Extending Below the Inguinal Ligament for Treatment of Chronic Iliofemoral Venous Obstruction. *Ann. Vasc. Surg.* **2021**, *75*, 259–266, doi:10.1016/j.avsg.2021.01.115.
