# Supplementary material for: Time-Driven Activity-Based Costing for Capturing the Complexity of Healthcare Processes: The Case of Deep Vein Thrombosis and Leg Ulcers
Source: Int J Environ Res Public Health. 2023 May 13;20(10):5817. doi: 10.3390/ijerph20105817 (PMC10218671; doi:10.3390/ijerph20105817)
Supplement: Supplementary file 1 [file ijerph-20-05817-s001.zip › Supplementary Table S3.pdf]

Supplementary Table S3. Costs for the personnel and timing for the different phases (timing of the different professionals may overlap in the single phase)*.

Role	Capacity cost rate (euro per minute, mean)	Time per phase (minutes)
**<u>Stenting process</u>**		
Administrative staff	0.57	1. Patient reception (14′)
Nurse	0.60	1. Patient reception (3′)<br>2. Preliminary assessment (21′)<br>3. Pre-operative hospitalization (94′)<br>4. Preparation for surgery (2x35′)<br>5. Surgical Intervention (2x62′)<br>6. Surgery closure (54′)<br>7. Post-operative hospitalization (2x95′)<br>8. Post-operative assessment (35′)<br>9. Discharge (33′)
Carer	0.53	1. Patient reception (7′)<br>2. Preliminary assessment (5′)<br>3. Pre-operative hospitalization (94′)<br>4. Preparation for surgery (20′)<br>6. Surgery closure (10′)<br>7. Post-operative hospitalization (30′)
Vascular surgeon	1.55	2. Preliminary assessment (90′)<br>5. Surgical Intervention (2x75′)<br>6. Surgery closure (15′)<br>7. Post-operative hospitalization (10′)<br>8. Post-operative assessment (19′)<br>9. Discharge (37′)
Radiology technician	0.64	4. Preparation for surgery (30′)<br>5. Surgical Intervention (60′)<br>6. Surgery closure (15′)
Anesthetist	1.48	2. Preliminary assessment (20′)<br>5. Surgical Intervention (90′)<br>6. Surgery closure (15′)
**<u>SOC</u>**		
Administrative staff	0.37	1. First visit<br>- Patient reception (2′)<br>- Acquisition of informed consent (3′)<br><br>2. Subsequent visits and treatment (each month)<br>- Patient reception (2′)
Nurse	0.54	1. First visit (85′ - all sub-phases with the exception of “Patient reception” and “Acquisition of informed consent”)<br><br>2. Subsequent visits and treatment (each month) (167′, all sub-phases)<br><br>3. Final phase (in case of ulcer healing) (65′, all sub-phases)

Role	Capacity cost rate (euro per minute, mean)	Time per phase (minutes)
Vascular surgeon	1.10	1. First visit (64′, all sub-phases with the exception of "Patient reception" and "Acquisition of informed consent")<br><br>2. Subsequent visits and treatment (each month)<br>- visit, 20′<br>- treatment and bandages, 32′<br><br>3. Final phase (in case of ulcer healing)<br>(60′, all sub-phases)

* Values in Tables 1 and 2 are obtained by multiplying the capacity cost rate and the time per phase for the different professionals involved (results may be slightly different due to rounding)
