# Supplementary material for: Time-Driven Activity-Based Costing for Capturing the Complexity of Healthcare Processes: The Case of Deep Vein Thrombosis and Leg Ulcers
Source: Int J Environ Res Public Health. 2023 May 13;20(10):5817. doi: 10.3390/ijerph20105817 (PMC10218671; doi:10.3390/ijerph20105817)
Supplement: Supplementary file 1 [file ijerph-20-05817-s001.zip › Supplementary Table S4.pdf]

Supplementary Table S4. Parameters used in probabilistic sensitivity analysis.

Parameter	Baseline value	Parameters for one-way sensitivity analyses		Parameters for probabilistic sensitivity analysis			
		Lower limit	Upper limit	standard error of the mean	distribution	alpha/mean of logs	beta/lambda/standard error of logs
**Costs (EUR)**							
Cost for stenting procedure	5234.00	4187.20	6280.80	1046.80	Gamma	25.00	209.36
Cost first visit - active ulcer	243.86	195.09	292.63	48.77	Gamma	25.00	9.75
Cost compression therapy/anticoagulants (monthly)*	38.72	30.98	46.46	7.74	Gamma	25.00	1.55
Cost FUP - active ulcer (monthly)	406.11	324.89	487.33	81.22	Gamma	25.00	16.24
Cost final visit - healed ulcer	127.95	102.36	153.54	25.59	Gamma	25.00	5.12
Cost compression therapy/anticoagulants (final treatment)*	185.50	148.40	222.60	37.10	Gamma	25.00	7.42
**Quality of life**							
Utility active ulcer	0.730	0.58	0.88	0.15	Beta	6.02	2.23
Utility recurred ulcer	0.640	0.51	0.77	0.13	Beta	8.36	4.70
Utility healed ulcer	1.000	0.80	1.00				
**Outcomes**							
Stenting							
Stenting % ulcers healed	81%	0.65	0.97	0.16	Beta	3.94	0.92
Stenting mean healing time (months)	2	1.60	2.40	0.40	Gamma	25.00	0.08
Standard of care - SOC							
SOC % ulcers healed	61%	0.49	0.73	0.12	Beta	9.14	5.84
SOC mean healing time (months)	3	2.40	3.60	0.60	Gamma	25.00	0.12
**Other parameters**							
Discount rate	3%	2.4%	3.6%				

* cost borne by the patient
