# Supplementary figures and images for: Time-Driven Activity-Based Costing for Capturing the Complexity of Healthcare Processes: The Case of Deep Vein Thrombosis and Leg Ulcers
Source: Int J Environ Res Public Health. 2023 May 13;20(10):5817. doi: 10.3390/ijerph20105817 (PMC10218671; doi:10.3390/ijerph20105817)

**Supplementary Figure S4.** Forest plots for pooled % of procedural success for stenting.

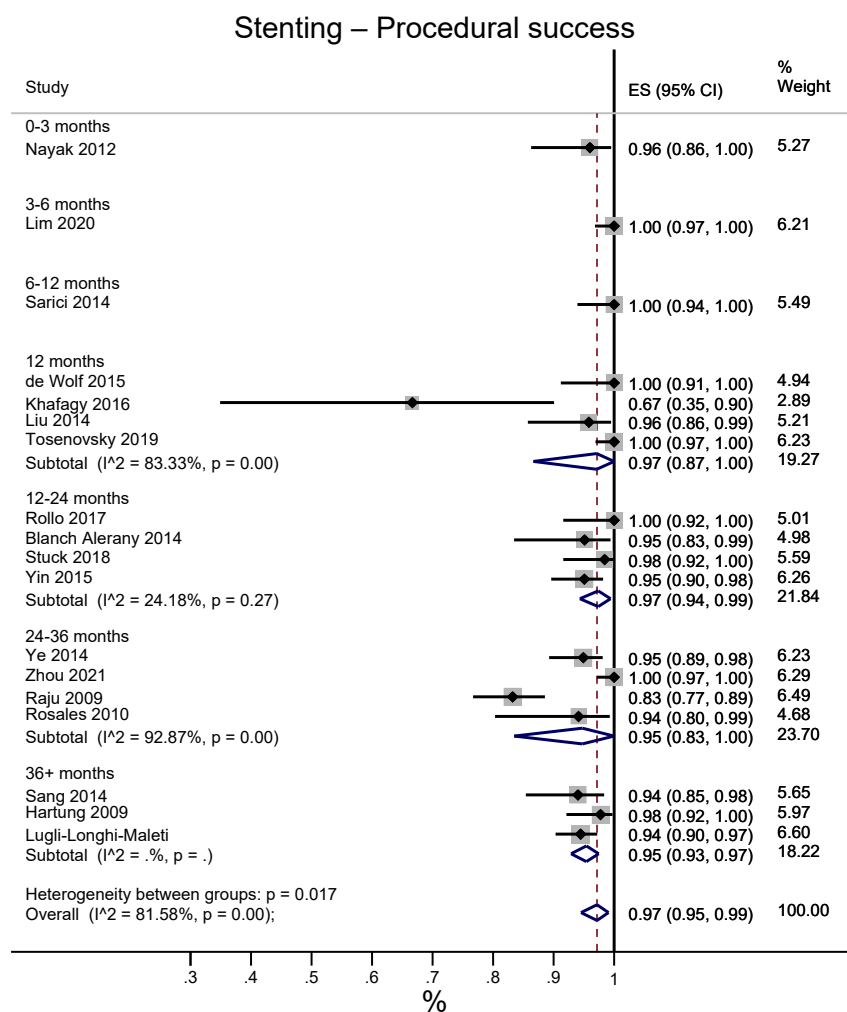

Supplement: Supplementary file 1 [file ijerph-20-05817-s001.zip › Supplementary Figure S4.pdf]
